# Patient-Derived Gastric Cancer Assembloid Model Integrating Matched Tumor Organoids and Stromal Cell Subpopulations

**DOI:** 10.3390/cancers17142287

**Published:** 2025-07-09

**Authors:** Irit Shapira-Netanelov, Olga Furman, Dikla Rogachevsky, Galia Luboshits, Yael Maizels, Dmitry Rodin, Igor Koman, Gabriela A. Rozic

**Affiliations:** 1 Institute for Personalized and Translational Medicine, Department of Molecular Biology, Ariel University, Ariel 4070000, Israel; iritsh@ariel.ac.il (I.S.-N.); olgaf@ariel.ac.il (O.F.); galialu@ariel.ac.il (G.L.); igorko@ariel.ac.il (I.K.); 2 Independent Researcher, Ariel 4070000, Israel; diklarog@gmail.com (D.R.); yael.maizels@gmail.com (Y.M.); rodin.dmitry@icloud.com (D.R.)

**Keywords:** assembloids, organoids, stromal cell subpopulations, gastric cancer, drug resistance, tumor microenvironment, personalized medicine

## Abstract

**Background/Purpose**: Conventional three-dimensional in vitro tumor models often fail to fully capture the complexity of the tumor microenvironment, particularly the diverse populations of cancer-associated fibroblasts that contribute to poor prognosis and treatment resistance. The purpose of this study is to develop a patient-specific gastric cancer assembloid model that integrates tumor epithelial cells with matched stromal cell subtypes, each derived using tailored growth media to enhance cancer preclinical research and advance personalized therapeutic strategies. **Methods**: Tumor tissue was dissociated, and cells expanded in media for organoids, mesenchymal stem cells, fibroblasts, or endothelial cells. The resulting tumor-derived subpopulations were co-cultured in an optimized assembloid medium supporting each cell type’s growth. Biomarker expression was assessed by immunofluorescence staining, and transcriptomic profiles were analyzed by RNA sequencing. Drug responsiveness was evaluated using cell viability assays following treatment with various therapeutic agents. **Results**: The optimized co-culture conditions yielded assembloids that closely mimicked the cellular heterogeneity of primary tumors, confirmed by the expression of epithelial and stromal markers. Compared to monocultures, the assembloids showed higher expression of inflammatory cytokines, extracellular matrix remodeling factors, and tumor progression-related genes across different organoids and stromal ratios. Drug screening revealed patient- and drug-specific variability. While some drugs were effective in both organoid and assembloid models, others lost efficacy in the assembloids, highlighting the critical role of stromal components in modulating drug responses. **Conclusions**: This assembloid system offers a robust platform to study tumor–stroma interactions, identify resistance mechanisms, and accelerate drug discovery and personalized therapeutic strategies for gastric cancer.

## Simple Summary

Organoid models often fail to capture the complex niche of patient-specific tumors. In this study, we present a novel methodology for generating gastric cancer assembloids composed of matched tumor organoids and stromal cell subpopulations, which closely recapitulate the cellular heterogeneity and microenvironment of primary tumors. The inclusion of autologous stromal cell subpopulations significantly influences gene expression and drug response sensitivity. By incorporating diverse stromal cell populations derived from the same tumor tissue as the organoids, these assembloids enable a more comprehensive investigation of individual tumor biology, biomarker expression, transcriptomic profiles, and cell–cell interactions. The model also supports personalized drug screening and the optimization of combination therapies. Altogether, the integration of patient-specific stromal cell subsets enhances the physiological relevance of preclinical testing, providing insights into resistance mechanisms and ultimately contributing to the development of more effective therapeutic strategies.

## 1. Introduction

Gastric cancer is the fifth most diagnosed carcinoma and the second leading cause of cancer-related deaths worldwide. For patients with locally advanced, unresectable, or metastatic disease, the five-year survival rate remains below 10% despite available treatments such as surgery, chemotherapy, radiotherapy, targeted therapy, and immune-based therapy [1]. This poor prognosis is partly attributed to the significant heterogeneity of gastric tumors, leading to variable treatment responses and clinical outcomes [2,3].

Current personalized treatment approaches focus on identifying druggable mutations in patient tumor cells. However, the clinical benefit of matched targeted therapies remains limited. Only a few FDA-approved drugs are available for gastric cancer, including trastuzumab for HER2-positive tumors and ramucirumab for VEGFR2-positive subtypes [4,5]. These limitations highlight the need for better predictive models and more effective use of targeted therapies, including those approved for other cancer types.

Patient-derived gastric cancer organoids (PDGCOs) have emerged as promising ex vivo models that closely mimic the heterogeneous morphology, genomic landscape, and molecular characteristics of gastric tumors [6,7,8,9]. However, most organoids models lack a comprehensive representation of the tumor microenvironment, which comprises diverse stromal cell types, including mesenchymal stem cells, cancer-associated fibroblasts (CAFs), endothelial cells, adipocytes, immune cells, as well as the surrounding extracellular matrix. The composition of stromal cells evolves as the tumor progresses, and microenvironment-based tumor classifications have shown prognostic value in gastric cancer [10,11,12,13,14], with high stromal cells infiltration linked to poor prognosis and reduced therapeutic efficacy, especially immunotherapy [15,16].

Among stromal components, CAFs are particularly prominent, constituting up to 70% of the tumor stroma and playing crucial roles in disease progression and treatment resistance [17]. CAFs influence tumor behavior through direct interaction and paracrine signaling [14,18,19]. Mechanistically, CAFs contribute to drug resistance by secreting exosomes and extracellular matrix components, which form physical barriers and alter drug sensitivity [20,21,22]. This effect has been documented for agents like 5-fluouracil [23,24], oxaliplatin [25], cisplatin [26], and paclitaxel [27].

In addition, patients with gastric cancer exhibit increased peripheral trafficking of mesenchymal stem cells compared to healthy individuals, with these cells preferentially accumulating within the tumor niche [28]. Tumor-associated mesenchymal stem cells exert pro-tumorigenic effects in gastric cancer, actively contributing to tumor progression and aggressiveness [29,30,31] by promoting epithelial-to-mesenchymal transition, angiogenesis, migration, and invasion [32,33].

Despite growing evidence on the importance of stromal cell diversity in gastric cancer progression and treatment resistance, integrating these elements into patient-derived organoid models remains challenging. In this study, we developed a robust method to generate three-dimensional (3D) patient-derived gastric cancer assembloids (PDGCAs) by combining patient-matched stromal subpopulations with PDGCOs. We compared the assembloids to the original tumor in terms of cellular composition and evaluated their responsiveness to chemotherapy and targeted therapies. This integrated model captures key tumor–stroma interactions and provides a physiologically relevant platform for identifying therapeutic vulnerabilities in gastric cancer.

## 2. Materials and Methods

### 2.1. Establishment of PDGCOs and Stromal Cells Cultures

Human gastric cancer and adjacent normal tissues were obtained from consenting patients (IRB approval MID-054-2019, protocol No. IPTM-3D). Tumor tissue was divided as follows: one-third frozen for DNA isolation, one-third fixed in 4% paraformaldehyde (Electron Microscopy Sciences, Hatfield, PA, USA) for immunofluorescence, and the remaining used for cell culture. Tissue digestion was performed using 1 mg·mL^−1^ Collagenase I (Gibco), 0.26 U·mL^−1^ Liberase (Roche, Basel, Switzerland) and 10 µg·mL^−1^ DNAse (Roche), followed by separation into organoid and stromal cell cultures. PDGCOs were cultured as previously described [6,7,8,9,34] with Advanced DMEM/F12 (Gibco, Waltham, MA, USA) supplemented with 10 mM HEPES (Gibco), 2 mM GlutaMAX (Gibco), 100 U·mL^−1^ penicillin/streptomycin (Gibco), 1.25 mM N-acetylcysteine (Biogems, Westlake Village, CA, USA), 1× B27 (Gibco), 500 ng·mL^−1^ R-spondin (Peprotech, Cranbury, NJ, USA), 100 ng·mL^−1^ noggin (Peprotech), 100 ng·mL^−1^ Wnt3A (Peprotech), 10 nM gastrin (Biogems), 100 ng·mL^−1^ IGF1 (Peprotech), 10 ng·mL^−1^ FGF2 (Peprotech), 10 ng·mL^−1^ FGF10 (Peprotech), 50 ng·mL^−1^ EGF (Peprotech), 1 µM prostaglandin E2 (Peprotech), 5 µM SB202190 (Apexbio, Houston, TX, USA), A83-01 (Biogems), and 4 mM nicotinamide (Sigma, St. Louis, MO, USA). After the initial seeding and after passaging, 10 μM Y-27632 (Peprotech) was added, with media replaced twice weekly. Stromal cells were maintained in mesenchymal stem cell medium (MSCM, ScienCell, Carlsbad, CA, USA), endothelial cell medium (ECM, ScienCell), or fibroblast growth medium (FM2, ScienCell). Cells were cryopreserved or subcultured using Accutase (Millipore, Burlington, MA, USA). Cells were incubated at 37 °C, 5% CO_2_. Brightfield images were captured using an Olympus CKX53e microscope. Technical consistency was maintained by processing fresh tumor samples from different sources under identical conditions.

### 2.2. Establishment of PDGCAs Co-Cultures

PDGCOs were dissociated mechanically, and stromal cells were dissociated with Accutase. A total of 3000 dissociated cells were combined in different ratios and seeded in an assembloid medium on 384-well, flat-bottom, low-attachment plates (Greiner Bio-One, Kremsmünster, Austria). The assembloid medium consisted of Advanced DMEM/F12 supplemented with 10 mM HEPES, 2 mM GlutaMAX, 100 U·mL^−1^ penicillin/streptomycin, 1× non-essential amino acids (Biological Industries, Kibbutz Beit Haemek, Israel), 1× sodium pyruvate (Biological Industries), 1.25 mM N-acetylcysteine, 1× B27, 500 ng·mL^−1^ R-spondin, 100 ng·mL^−1^ noggin, 100 ng·mL^−1^ Wnt3A, 10 nM gastrin, 100 ng·mL^−1^ IGF1, 10 ng·mL^−1^ FGF2, 10 ng·mL^−1^ FGF10, 50 ng·mL^−1^ EGF, 1 µM prostaglandin E2, 50 µg·mL^−1^ ascorbic acid (Sigma), 5 ng·mL^−1^ VEGF (Peprotech), 20 µg·mL^−1^ heparin (Peprotech), 5 μM Y-27632, 5% fetal bovine serum (Gibco), 10 ng·mL^−1^ fibronectin, and 5% matrigel. Half of the medium was replenished every 3–4 days. The co-cultures were incubated at 37 °C, 5% CO_2_ on a shaker at 100 rpm. Brightfield images were captured using the Operetta CLS High-Content Analysis System (PerkinElmer, Waltham, MA, USA) at defined time points . To assess structural growth over time, the long axis of assembloids and organoids was measured using ImageJ software (version 1.53t, National Institutes of Health, Bethesda, MD, USA).

### 2.3. Multi-Dye Cell Staining of Dissociated Cells

Cells cultured in MSCM and ECM media were labeled with the CellTrace™ Violet Dye (Gibco), while cells in FM2 medium and PDGCOs were stained with the CellTrace™ CFSE (Gibco) or Minclaret CellVue^®^ far-red fluorescent kit (Sigma). Imaging was performed using an Olympus Fluoview FV3000 confocal microscope (Olympus, Tokyo, Japan).

### 2.4. Cell Viability Assay

Cell viability was assessed using the CellTiter-Glo kit (Promega, Madison, WI, USA). PDGCAs and PDGCOs were cultured for 48 h before exposure to drug treatments for 72 h, including FLOT (comprising 7.5 µM 5-fluorouracil (ApexBio), 0.8 µM leucovorin (ApexBio), 3.5 µM oxaliplatin (ApexBio), and 5 µM docetaxel (ApexBio)), FOLFIRI (comprising 7.5 µM 5-fluorouracil (ApexBio), 0.8 µM leucovorin (ApexBio), 3.5 µM oxaliplatin (ApexBio), 5 µM irinotecan (ApexBio)), 5 µM paclitaxel (ApexBio), 1 µM crizotinib (ApexBio), 0.8 µM osimertinib (TargetMol, Wellesley Hills, MA, USA), 0.150 µM afatinib (ApexBio), 8 μM capmatinib (TargetMol), 0.180 µM dasatinib (ApexBio), 4 µM imatinib (ApexBio), 0.4 µM palbociclib (ApexBio), and 3 µM gefitinib (ApexBio). Drug concentrations were based on plasma levels [35]. Luminescence was measured using a CLARIOstar microplate reader (BMG Labtech, Ortenberg, Germany). Alternatively, cell viability was assessed using a Calcein-AM/Ethidium Homodimer-1 double-staining assay (both from Invitrogen, Waltham, MA, USA). After drug treatment, PDGCAs and PDGCOs were incubated with 5 μM Calcein-AM and 4 μM Ethidium Homodimer-1 in DPBS for 40 min at 37 °C. Fluorescence images were acquired using an Olympus Fluoview FV3000 confocal microscope. Live cells exhibited green fluorescence, while dead cells were visualized with red fluorescence.

### 2.5. Immunofluorescence

Samples were fixed in 4% paraformaldehyde and embedded in 2% agarose gel (BioRad, Hercules, CA, USA) or paraffin. Antigen retrieval was performed using Tinto DeepParaffinator-Citrate (Bio SB, Goleta, CA, USA). After permeabilization and blocking with TrueBlack IF buffer (Biotium, Fremont, CA, USA), samples were incubated overnight with primary antibodies: 1:100 anti–CD146 (Biotechne R&D systems, Santa Clara, CA, USA), 1:100 anti-EPCAM (Santa Cruz, Dallas, TX, USA), 1:100 anti-ALDH1A1 (LsBio, Seattle, WA, USA), 1:200 anti-CD133 (Novus Biologicals, Englewood, CO, USA), 1:350 anti-CDH1 (Novus Biologicals), 1:200 anti-Vimentin (Abcam, Boston, MA, USA), 1:1000 anti–α-SMA (Abcam), 1:600 anti-CD105 (ThermoFisher Scientific, Waltham, MA, USA), and 1:350 anti-CD73 (Abcam). Samples were incubated with secondary antibodies (Jackson Immunoresearch, West Grove, PA, USA) for 1 h. Afterwards, 10 ng. mL-1 Hoechst (Sigma) was used for nuclear counterstaining. Samples were mounted in EverBrite TrueBlack Hardset mounting medium (Biotium) and imaged with an Olympus Fluoview FV3000 confocal microscope.

### 2.6. Oil Red O Staining

Intracellular lipids in FM2-cultured stromal cells grown in 24-well plates were detected by Oil Red O staining (Sigma). Cells (~80% confluence) were fixed with 4% paraformaldehyde for 15 min, rinsed, and incubated in 60% isopropanol for 5 min. Cells were then stained with 0.3% Oil Red O solution for 15 min, washed with water, and examined by brightfield microscopy. Red cytoplasmic inclusions indicated the presence of neutral lipid droplets.

### 2.7. Enzyme-Linked Immunosorbent Assay (ELISA)

IL-8 and MMP1 concentrations in the conditioned medium of organoids and assembloids were measured using commercially available ELISA kits—Human IL-8 ELISA Kit (Abcam, ab214030) and Human MMP-1 ELISA Kit (Abcam, ab100604)—following the manufacturer’s instructions. Supernatants were collected after 72 h, cleared by centrifugation, and analyzed in duplicate. Absorbance was measured at 450 nm, and cytokine levels were calculated using standard curves generated with kit-provided standards.

### 2.8. Whole-Exome Sequencing and Bioinformatic Analysis

DNA was extracted using the Allprep DNA/RNA kit (Qiagen, Hilden, Germany). Whole-exome sequencing was performed on gastric cancer tumors, normal tissues, PDGCOs, and stromal cells cultured in FM2, ECM, and MSCM media, outsourced to BGI Genomics Co., Ltd. (Shenzhen, China), using the DNBSEQ-G400 platform. Variant calling was performed using the GATK HaplotypeCaller (v4.0.11). Somatic mutations were identified using the COSMIC database [36] and mutation pathogenicity was classified using ClinVar [37]. Pathway analysis was performed with the WebGestalt Toolkit 2019 [38], and significant pathways (False Discovery Rate (FDR) q-value < 0.05) were visualized in Python version 3.11.8.

### 2.9. RNA Sequencing and Differential Gene Expression Analysis

RNA was extracted using the Allprep DNA/RNA Kit. RNA sequencing was conducted on PDGCOs, stromal cells cultured in FM2, ECM, and MSCM media, and PDGCAs with various cell ratios. RNA sequencing and data analysis were outsourced to The Nancy and Stephen Grand Israel National Center for Personalized Medicine (Rehovot, Israel). Libraries were prepared and sequenced on an Illumina instrument (Illumina, San Diego, CA, USA), yielding approximately 26 million reads per sample. Reads were mapped to the GRCh38 using STAR version STAR-2.7.3a [39], and differential expression was analyzed with DESeq2 [40]. *p*-values were adjusted using Benjamini and Hochberg [41]. The analysis pipeline was executed with snakemake [42]. Pathway analysis of differentially expressed genes (|log2FoldChange| ≥ 1, *p* ≤ 0.05, *n* ≥ 30) was performed with WebGestalt Toolkit [38], with significant pathways (FDR q-value < 0.05) visualized in Python.

### 2.10. Statistical Analysis

Statistical significance between any two groups was analyzed using unpaired two-tailed Student’s *t*-tests. For multiple groups, ANOVA was followed by Tukey’s Honest Significant Difference test. Significance levels were as follows: * *p* < 0.05, ** *p* < 0.01, *** *p* < 0.005, **** *p* < 0.001. Data are presented as mean ± SEM.

## 3. Results

### 3.1. PDGCOs and Stromal Cells Preserve Cellular Composition and Mutational Spectrum of Primary Tumor Tissue

**Figure 1:**
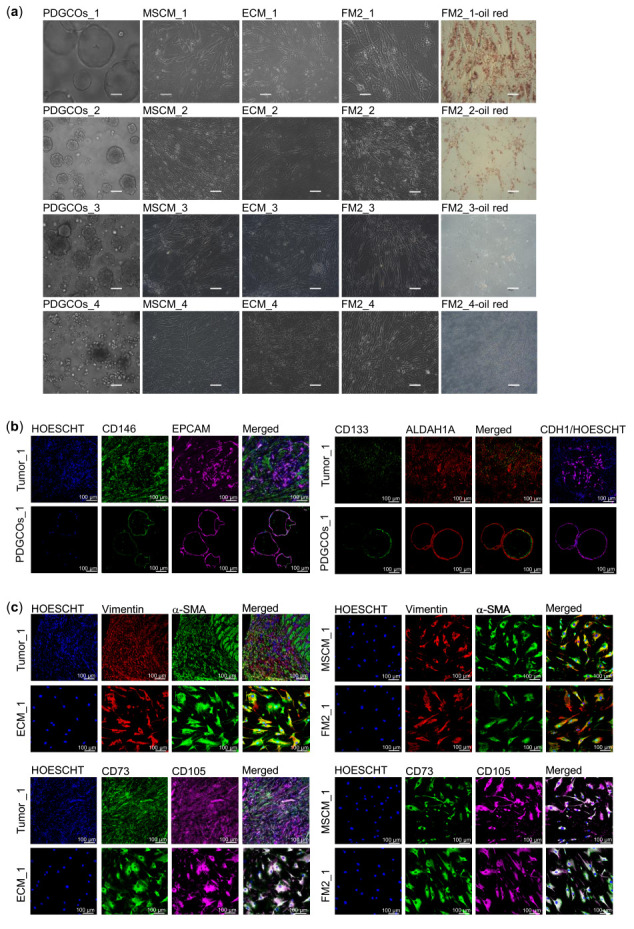
Characterization of gastric tumor-derived PDGCOs and autologous stromal cell subpopulations. (**a**) Representative brightfield images of matched PDGCOs and stromal cell subpopulations cultured in mesenchymal stem cell medium (MSCM), endothelial cell medium (ECM), and fibroblast medium (FM2), labeled by patient (1–4). Also shown are FM2-cultured cells stained with Oil Red.Scale bar = 100 µm (**b**) Representative immunofluorescence images of PDGCOs and matched tumor tissue stained for epithelial and stemness markers: CD146/EPCAM, ALDH1A1/CD133, or CDH1. Nuclei were counterstained with Hoechst. (**c**) Representative immunofluorescence images of stromal cells cultured in MSCM, ECM, or FM2, as well as paired tumor tissue, stained for stromal and mesenchymal markers: Vimentin/αSMA or CD105/CD73. Nuclei were counterstained with Hoechst. Scale bar = 100 µm.

To develop an ex vivo gastric cancer model, tumor-dissociated cells were cultured in media optimized for organoids, mesenchymal stem cells, endothelial cells, or fibroblast cells to preserve both epithelial and stromal components (Figure 1a). Brightfield imaging showed organoids forming glandular structures or solid clusters, while stromal cells adhered in a monolayer with fusiform morphology, indicative of mesenchymal characteristics. FM2-cultured stromal cells from specific tumor samples (FM2_1 and FM2_2) displayed prominent vacuole-like structures, suggesting metabolic heterogeneity within the stromal population. Oil Red O staining confirmed the lipidic nature of these vacuoles, with strong staining indicating the accumulation of neutral lipids and supporting the presence of lipid-rich compartments in FM2 cultures.

Immunofluorescence confirmed that PDGCOs expressed the epithelial markers EPCAM and CDH1, along with the stemness markers ALDH1A and CD133 (Figure 1b). Stromal cells expressed the canonical mesenchymal stromal markers, including Vimentin, the CAF marker αSMA, and mesenchymal stem cell markers CD105 and CD73, across all culture conditions, reflecting the cellular phenotypes observed in the original tumor tissue (Figure 1c, Supplementary Figure S1). Although all stromal subpopulations were consistently isolated across patient samples, some interpatient variability was observed in morphological features, such as lipid vacuole presence, likely reflecting biological heterogeneity rather than technical variation.

**Figure 2:**
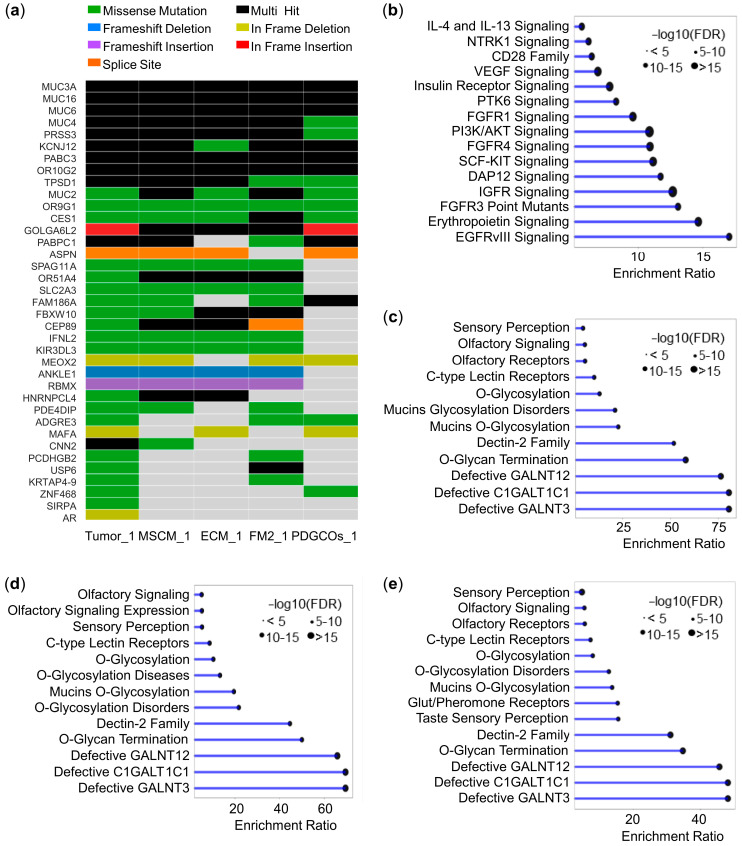
Comparative genomic landscape of gastric tumor-derived PDGCOs and matched stromal cells. (**a**) Oncoplot showing the distribution and types of somatic mutations associated with gastric adenocarcinoma across tumor tissue and tumor-derived cultured cells. Mutation types are indicated by color: missense (dark green), frameshift deletion (blue), frameshift insertion (violet), splice site (orange), multi-hit (black), in-frame deletion (light green), and in-frame insertion (red). (**b**–**e**) Reactome and KEEG enrichment analysis of pathways affected by mutations in (**b**) normal stomach tissue, (**c**) tumor tissue, (**d**) PDGCOs, and (**e**) tumor-derived stromal cells cultured in MSCM.

Whole-exome sequencing of a representative sample identified 37 non-pathogenic gastric cancer-related somatic mutations. These mutations were unevenly distributed between PDGCOs and the different stromal cell subpopulations, highlighting their genetic similarity with the parental tumor (Figure 2a).

Functional analysis of the mutational landscape revealed that germline mutations were associated with cancer susceptibility and key signaling pathways involving tyrosine kinase receptors, growth factor receptors, and PI3K-AKT signaling. In contrast, somatic mutations were primarily enriched in genes linked to glycosylation, mucin pathways, and olfactory signaling across tumor tissue, PDGCOs, and stromal cell subpopulations (Figure 2b–e). These results further support the genetic fidelity of the different cultured cell subsets.

### 3.2. Cultured Stromal Cell Subpopulations in MSCM, FM2, and ECM Exhibit Differential Gene Signatures

**Figure 3:**
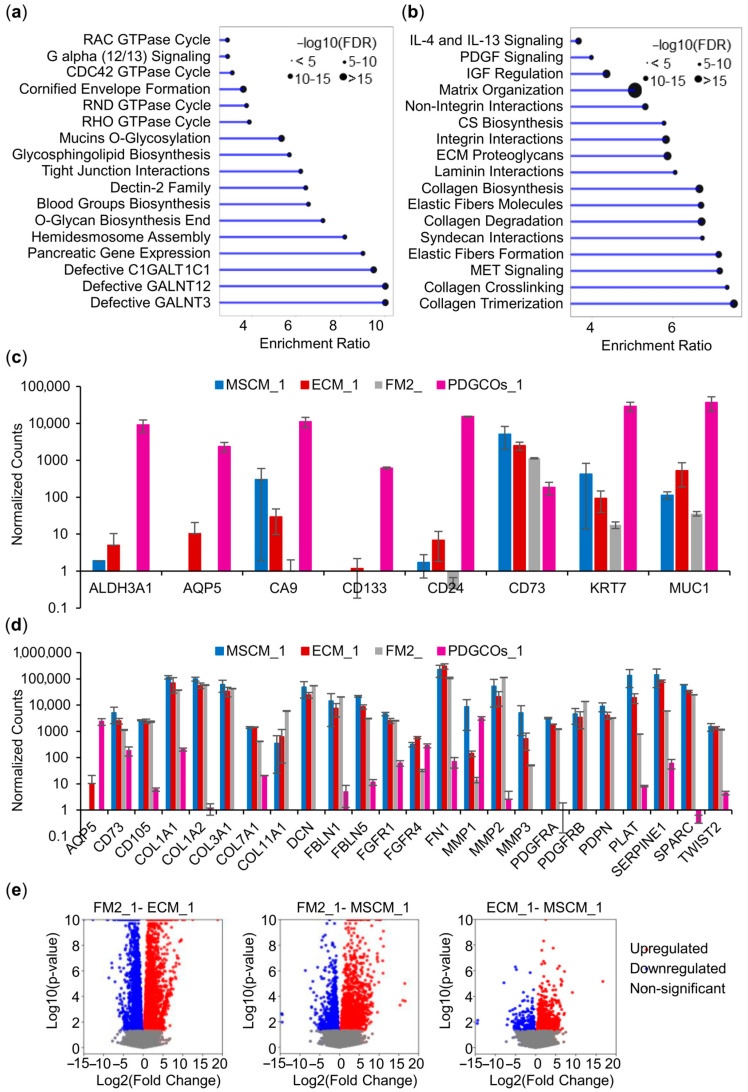
Gene expression analysis of gastric tumor PDGCOs and matched stromal cells. (**a**) Lollipop chart of Reactome and KEEG pathway enrichment for genes upregulated in PDGCOs compared to stromal cells. (**b**) The lollipop chart showing pathways enriched in stromal cells compared to PDGCOs. (**c**) Bar plot comparing expression of epithelial and (**d**) mesenchymal markers in PDGCOs and stromal cell cultures. (**e**) Volcano plot displaying differentially expressed genes in stromal cells cultured in FM2 compared to ECM and MSCM, as well as ECM compared to MSCM media. Data are presented as mean ± SEM.

RNA sequencing of PDGCOs and cancer-associated stromal cells from a representative patient (PDGO_1) identified 2340 differentially expressed genes with 1195 upregulated and 1145 downregulated PDGCOs_1 compared to stromal cells. PDGCOs showed higher expression of genes related to the Rho family of small GTPases signaling (RHO, RAC, CDC42, RND), as well as mucin glycosylation and cell junction formation (Figure 3a). In contrast, stromal cells displayed elevated expression of genes involved in extracellular matrix organization (collagen synthesis and degradation, elastic fiber formation, integrin cell surface interactions), along with interleukin, PDGF, and MET signaling pathways (Figure 3b).

Transcriptomic profiling revealed that PDGCOs_1 expressed elevated levels of tumor epithelial and stem cell markers, such as EPCAM, CDH1, ALDH3A1, CA9, CD24, and CD133 (Figure 3c). Stromal cultures consistently expressed canonical CAF-related markers, including COL1A1, COL1A2, COL3A1, COL7A1, DCN, FGFR1, FN1, SPARC, PDGFRA, PDGFRB, and PDPN, confirming their fibroblast-like identity (Figure 3d). Notably, several markers, including CD73, FBLN5, FGFR4, MMP1, MMP3, PLAT, and SERPINE 1, were differentially expressed depending on the culture medium, indicating functional divergence among stromal subpopulations.

Further transcriptomic analysis revealed that cells cultured in ECM, FM2, and MSCM exhibited distinct gene expression profiles consistent with a heterogeneous stromal population (Figure 3e). FM2-cultured cells were enriched in lipid metabolism-related genes (cholesterol, terpenoid biosynthesis, steroids). In contrast, ECM- and MSCM-cultured cells were enriched in genes related to interleukins (IL-4R, IL-7R, IL-6, IL-17, IL-18, and IL-27RA), differentiation of T helper cells, HIF-1, NTRK1, and TNF signaling pathways, as well as TP53 and NGF transcription. These findings highlight the functional diversity of the stromal compartment and underscore the critical role of media composition in shaping stromal phenotypes when modeling tumor–stroma interactions.

### 3.3. Generation of PDGCAs from Matched PDGCOs and Stromal Cell Subpopulations

To create a 3D organotypic model that simulates the complex tumor microenvironment, both pre-cultured PDGCOs and stromal cell subtypes derived from the same tumor tissue were co-cultured under three conditions: without a scaffold, with low-concentration matrigel, or with a combination of low-concentration matrigel and fibronectin scaffolds. The co-culture medium was based on the PDGCOs growth medium, excluding the inhibitors SB202190 and A83-01, and supplemented with components to support the growth of different stromal cell subpopulations, including VEGF, ascorbic acid, sodium pyruvate, fibronectin, heparin, and a low percentage of serum.

To evaluate the suitability of the co-culture (assembloid) medium, the viability of organoids and stromal cells, cultured separately in either organoid or assembloid media, was compared. Stromal cells exhibited improved viability and proliferation in the assembloid medium, while organoid viability and growth were similar in both media. Under low-attachment co-culture conditions, the assembloid medium supported the viability of both organoids and stromal cells. In contrast, assembloid viability was reduced in organoid medium (Figure S2).

**Figure 4:**
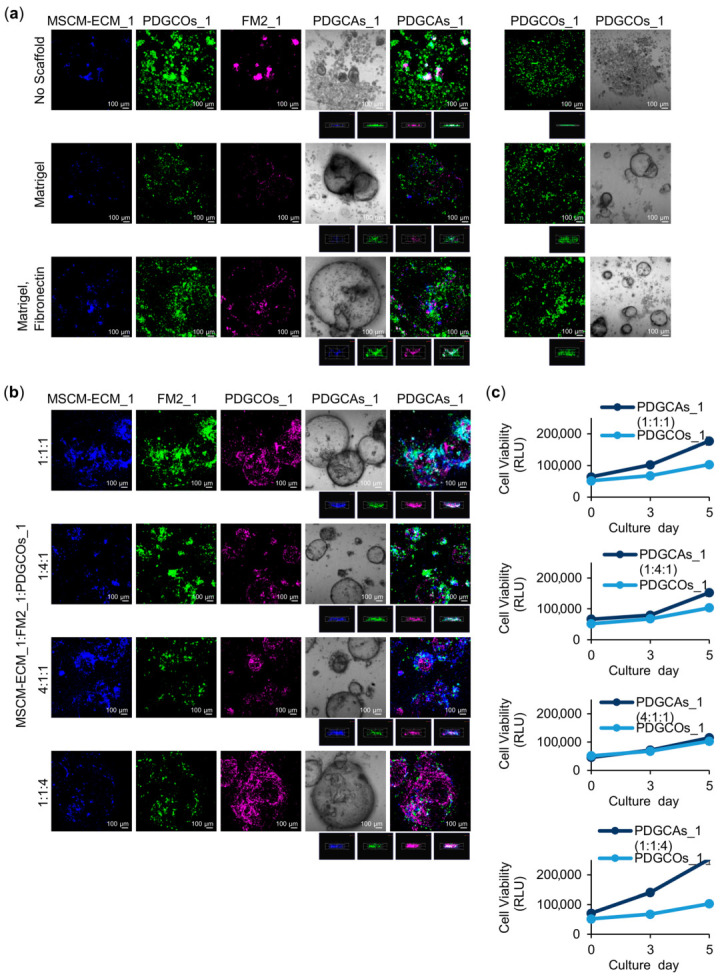
Generation of a gastric cancer assembloid model from matched PDGCOs and stromal cell subpopulations. Representative brightfield and Z-stacks immunofluorescence images of (**a**) PDGCAs composed of paired stromal cells cultured in MSCM-ECM (blue) and FM2 (magenta) media, along with PDGCOs (green), at a ratio of 1:1:4, under three conditions: no scaffold, matrigel, or matrigel with fibronectin. (**b**) PDGCAs composed of stromal cells cultured in MSCM-ECM (blue) and FM2 (green) media, as well as PDGCOs (magenta), at different ratios (1:1:1, 1:4:1, 4:1:1, or 1:1:4). (**c**) Viability of PDGCOs and PDGCAs across different ECM:FM2:PDGCOs ratios. Scale bar = 100 µm. Images acquired using a Zeiss confocal microscope (Zeiss, Oberkochen, Germany). Data are presented as mean ± SEM.

Multi-color staining enabled the identification of individual cell populations within the assembloid system (Figure 4a,b). Although this method enabled early tracking of individual populations, fluorescence intensity decreased over time due to dye dilution during cell proliferation, leaving some regions within larger structures unlabeled.

Without a scaffold, ECM:FM2: PDGCOs co-cultures remained separate and failed to form a cohesive 3D structure. In contrast, the addition of matrigel and fibronectin promoted cell aggregation, forming PDGCAs comprising multiple cell types that organized into larger organoid-like 3D structures. While the spatial organization of cells appeared similar between matrigel alone and matrigel supplemented with fibronectin, the presence of fibronectin was associated with a higher number of FM2-cultured cells. To evaluate the impact of initial cell composition on growth dynamics, we seeded the cells at different ratios. The proportions of different cell populations remained consistent with the initial seeding ratios after 5 days of culture (Figure 4b).

Assembloid growth was assessed using the CellTiter-Glo assay. PDGCAs containing higher proportions of PDGCOs, particularly at ECM:FM2:PDGCOs ratios of 1:1:1 and 1:1:4, exhibited faster growth compared to other ratios and PDGCOs monocultures (Figure 4c). To complement this viability data, assembloid diameters were measured throughout the culture period as a direct indicator of growth. As shown in Figure S3, assembloids across different ECM:FM2:PDGCOs ratios exhibited a significant increase in size over time, confirming active expansion of the 3D structures. These findings underscore the critical role of cellular composition in shaping assembloid growth dynamics.

### 3.4. PDGCAs Exhibit Biomarkers of Both PDGCOs and Stromal Cells and Promote the Expression of Immune-Related Genes

**Figure 5:**
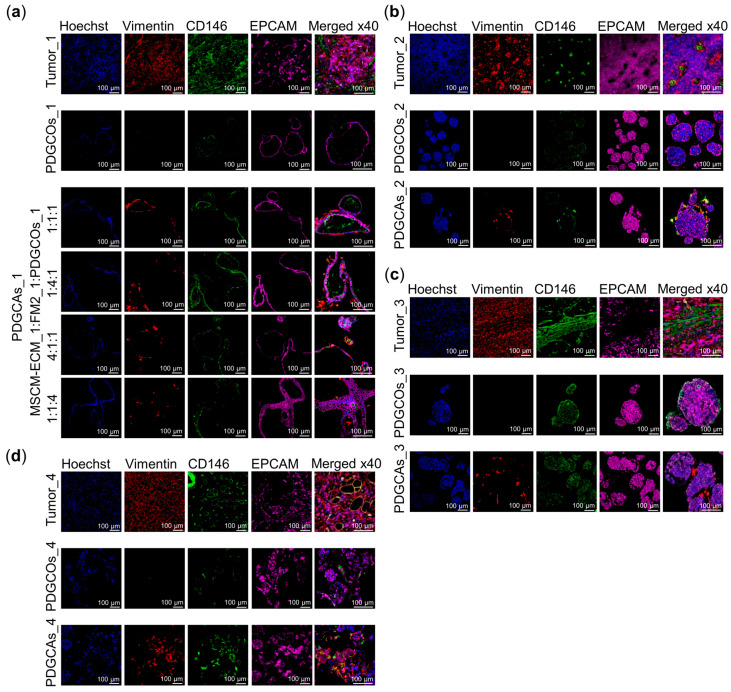
Protein visualization of epithelial and stromal cell markers in PDGCAs. (**a**–**d**) Representative Z-stack immunofluorescence images showing expression of Vimentin, CD146, and EPCAM in primary tumor tissue, PDCGOs, and PDCGAs from multiple patients. Nuclei were counterstained with Hoechst. Scale bar = 100 µm. Images acquired using a Zeiss confocal microscope.

To more accurately assess cellular composition and spatial organization, immunofluorescence staining was performed using lineage-specific markers (EPCAM for epithelial cells and Vimentin for stromal cells) along with nuclear counterstaining (Figure 5). PDGCAs_1 models were established using varying ECM:FM2:PDGCOs cell ratios, whereas PDGCAs_2-4 were generated using a fixed 1:1:4 ratio. This ratio was selected due to its higher tumor-to-stroma proportion, faster structural organization, and superior overall growth. Across all ratios and samples, assembloids consistently exhibited a characteristic spatial arrangement, with epithelial cells forming central clusters surrounded by stromal cells. Immunostaining confirmed the presence of both epithelial (EPCAM+) and stromal (Vimentin+) populations in all PDGCAs, mirroring the cellular composition of the native tissue (Figure 5). In contrast, PDGCOs alone showed minimal Vimentin expression, reflecting the absence of stromal components.

**Figure 6:**
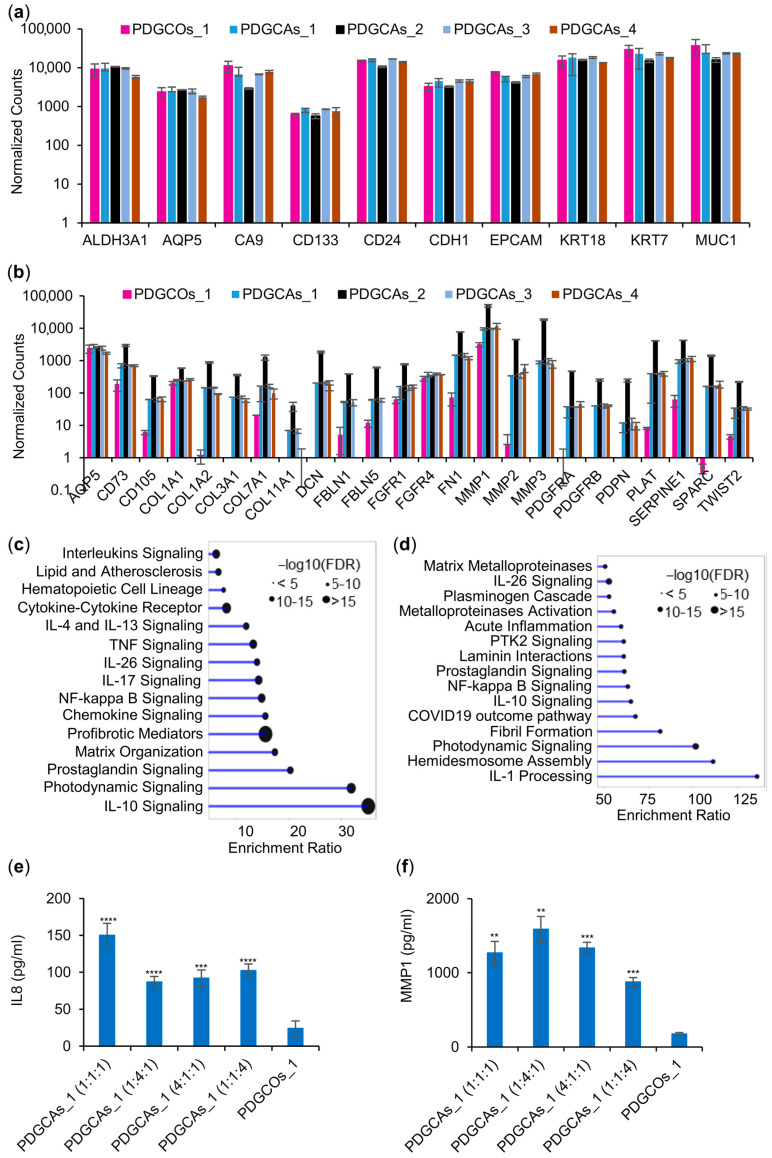
Effect of microenvironment composition on PDGCAs transcriptomic signatures. Bar plot of differentially expressed (**a**) epithelial and (**b**) mesenchymal markers in PDGCOs_1 and PDGCAs_1 at various ECM_1:FM2_1:PDGCO_1s ratios (1:1:1 (1), 1:4:1 (2), 4:1:1 (3), or 1:1:4 (4)). Lollipop chart of enriched signaling pathways identified in representative PDGCAs_1 cultured at ECM_1:FM2_1:PDGCOs_1 ratios of (**c**) 1:1:1 or (**d**) 1:4:1. Expression levels of IL-8 (**e**) and MMP-1 (**f**) in conditioned media from PDGCAs_1 and PDGCOs_1 cultures, quantified using ELISA. Data are presented as mean ± SEM. Statistical significance: ** *p* < 0.01, *** *p* < 0.005, **** *p* < 0.001. Comparisons: PDGCAs versus PDGCOs_1 (*).

Bulk RNA sequencing of the representative PDGCOs_1 and its matched PDGCAs_1 across the various ECM:FM2:PDGCOs ratios confirmed cellular heterogeneity. All PDGCAs expressed markers of tumor epithelial and stem cells (EPCAM, CDH1, KRT18, CA9, CD24, CD133), stromal and extracellular matrix components (COL3A1, COL5A1, DCN, PDGFRA, and PDGFRB), and mesenchymal stem cells (CD73, CD105, CD90) (Figure 6a,b).

Importantly, transcriptomic profiling confirmed that key gene expression patterns remained consistent across different cell ratios. Regardless of the ratio, PDGCAs consistently exhibited upregulation of genes involved in interleukin signaling (IL-1α, IL-6, IL-17, IL-23A, COX-2, CSF2, CSF3, TNF, CXCL3, CXCL5, PTGS2, SERPINB2), matrix metalloproteinases (MMP1, MMP3, MMP7, MMP9, MMP10, MMP24), and cancer-related pathways (BIRC3, BMP2, CXCL8, FGF2, FGFR3, HSP90AA1, TGFB3, WNT10A) (Figure 6c,d).

Regardless of cell ratios, PDGCAs consistently exhibited upregulation of genes involved in interleukin signaling (IL-1α, IL-1α, IL-6, IL-17, IL-23A, COX-2, CSF2, CSF3, TNF, CXCL3, CXCL5, PTGS2, SERPINB2), matrix metalloproteinases (MMP1, MMP3, MMP7, MMP9, MMP10, MMP24), and cancer-related pathways (BIRC3, BMP2, CXCL8, FGF2, FGFR3, HSP90AA1, TGFB3, WNT10A) (Figure 6c,d).

PDGCAs with a higher proportion of FM2-cultured cells relative to the other tested ratios showed increased expression of pro-inflammatory cytokines (IL-1α, IL-1β, IL-6, IL-11, IL-24, LIF), colony-stimulating factors (CSF2, CSF3), chemokines (CXCL8), and genes involved in inflammation and extracellular matrix remodeling (PTGS2, SERPINB2, SERPINB7, MMP1, MMP3, MMP10). Conversely, mucin genes (MUC5AC, MUCL3) were elevated in PDGCAs with a higher ECM-cultured cell ratio or in PDGCOs alone.

To validate the transcriptional data at the protein level, CXCL8 and MMP1 concentrations were measured in conditioned medium from assembloids and organoids using ELISA. Assembloids secreted significantly higher levels of both IL8 and MMP1 proteins (*p* < 0.001), irrespective of the epithelial-to-stromal cell ratio (Figure 6e,f). These results corroborate the RNA-seq findings and confirm that stromal components enhance immunosuppressive cytokine signaling in the co-culture system.

These findings highlight the role of stroma–epithelial interactions on the PDGCAs transcriptomic landscape, emphasizing how microenvironment composition influences cell communication, tissue organization, and tumor progression.

### 3.5. Impact of Stromal Cells on Tumor Response to Therapy: Insights from PDGCAs

To evaluate how stromal subpopulations influence chemotherapy response, we compared drug sensitivity in PDGCAs and PDGCOs after three days of treatment. In PDGCOs_1, FLOT, FOLFIRI, and paclitaxel reduced cell viability by ~50%. In contrast, the same treatments led to only a 20–30% reduction in PDGCAs_1, suggesting stromal-mediated resistance. Notably, this model was derived from a patient whose disease progressed despite receiving these drugs clinically.

**Figure 7:**
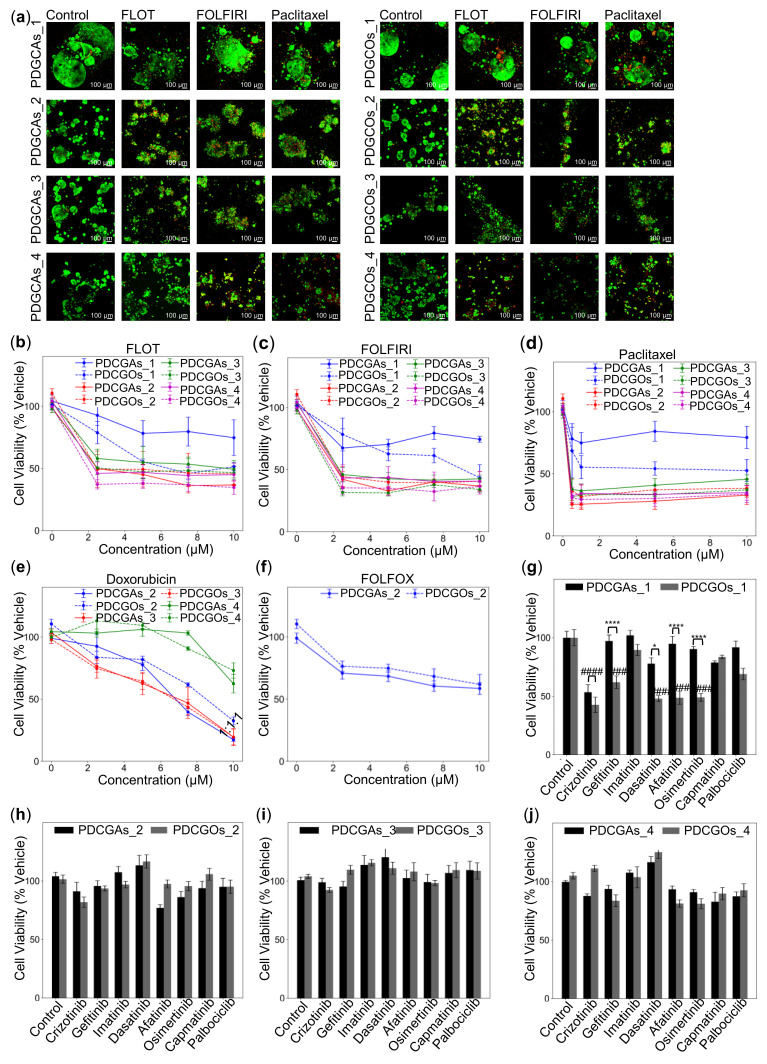
Therapy responsiveness in PDGCAs and matched PDGCOs. (**a**) Representative immunofluorescence images of paired PDGCAs and PDGCOs treated with FLOT, FOLFIRI, or Paclitaxel for 72 h, stained with Calcein (live cells, green) and Ethidium Homodimer-1 (dead cells, red). Scale bar = 100 µm. Images captured using Zeiss confocal microscope. (**b**–**f**) Cell viability of PDGCAs and PDGCOs after 72 h exposure to chemotherapies (FLOT, FOLFIRI, Paclitaxel, FOLFOX, or Doxorubicin), measured using CellTiter-Glo. (**g**–**j**) Cell viability assays evaluating the efficacy of targeted therapies across patient-derived PDGCAs and PDGCOs. Data represent mean ± SEM, (n = 3). Statistical significance: * *p* < 0.05, **** *p* < 0.001; ### *p* < 0.001; #### *p* < 0.0001. Comparisons: PDGCAs versus PDGCOs (*), treatment versus control (#). Samples 1–4 represent tumors derived from different patients.

Other patient-derived models showed significant cell death (*p* < 0.001) in response to treatment (Figure 7a–d). For example, PDCGOs_2 and PDGCAs_2 responded to FOLFOX, consistent with the patient’s favorable clinical outcome and absence of recurrence (Figure 7e). Similarly, PDCGOs_3 and PDGCAs_3 were sensitive to FLOT, which matched the patient’s neoadjuvant regimen. Both PDGCOs_2-3 and PDGCAs_2-3 responded to doxorubicin, whereas PDGCOs_4 and PDGCAs_4 were resistant (Figure 7f), reflecting inter- and intra-patient variability in drug response.

Although no imaging or detailed longitudinal clinical data were available to formally validate these findings, the observed concordance between patient responses and in vitro drug sensitivity supports the translational relevance of the model.

Targeted therapies showed variable efficacy. Crizotinib induced approximately 50% cell death in both PDGCOs_1 and PDGCAs_1 (*p* < 0.001). Similarly, dasatinib, afatinib, and osimertinib each reduced PDGCOs_1 viability by ~50% (*p* < 0.001), while gefitinib achieved a 37% reduction (*p* < 0.001). However, resistance increased in PDGCAs_1 following co-culture with stromal cells, increasing a stromal-driven mechanism (Figure 7g).

None of the targeted drugs reduced cell viability by more than 25% in PDGCOs_2-4 or PDGCAs_2-4 (Figure 7h–j). These findings underscore the role of stromal cells in modulating therapy resistance and highlight the importance of personalized PDGCAs models for guiding treatment decisions.

## 4. Discussion

Patient-derived tumor organoids have emerged as a powerful tool for disease modeling, drug discovery, and personalized medicine in cancer. They offer a system that bridges the gap between traditional in vitro cultures and in vivo models. However, they lack essential interactions with the tumor microenvironment, which plays a critical role in shaping cancer biology. This study introduces a 3D gastric cancer assembloid model that integrates autologous stromal cell subtypes with organoids, enabling dynamic tumor–stroma interactions through both direct cell contacts and paracrine signaling.

Assembloids, advanced 3D cultures combining epithelial cells and CAFs, have been used to study stromal interactions in models of malignant murine gastrointestinal cancers [43], human colon cancer [44], esophageal adenocarcinoma [45], bladder cancer [46], and lung cancer [47], with CAFs cultured in DMEM in most studies. In these systems, CAFs restore tumor-intrinsic signaling, improve the transcriptomic fidelity of organoids, and modulate drug sensitivity [48]. In gastric adenocarcinoma, co-cultures of patient-derived organoids with T-cells [49], dendritic cells [50], and other immune components have been described [51]. Recently, assembloids combining gastric tumor-derived CAFs cultured in DMEM with patient-derived xenograft organoids were shown to effectively replicate clinical drug responses [52]. In our model, assembloids are composed of matched PDGCOs and tumor stromal cells cultured in MSCM, FM2, or ECM media to generate distinct stromal cell subtypes that more accurately reflect the cellular diversity and complexity of the tumor microenvironment.

Gastric cancer exhibits high inter- and intra-tumoral heterogeneity, which contributes to tumor aggressiveness, therapeutic resistance, and variable clinical outcomes [53]. CAFs are themselves a heterogeneous population commonly classified into subtypes such as pro-inflammatory, vascular, matrix remodeling, and myofibroblastic CAFs, each playing distinct roles in shaping the tumor microenvironment [53,54,55,56]. In our study, stromal cells cultured in FM2 showed upregulation of genes related to lipid homeostasis and metabolic plasticity, suggesting that FM2 conditions promote adaptations characteristic of CAFs during tumor progression. This aligns with findings that, as cancer progresses, CAFs undergo lipidomic reprogramming, altering their metabolism, proliferation, and invasion [57,58], while cytokine secretion shifts towards an immunosuppressive microenvironment [59]. Correspondingly, PDGCAs_1 exhibited elevated transcriptomic expression of immunosuppressive signaling pathways and significantly higher secretion of IL8 and MMP1 compared to PDGCOs_1. These findings suggest that stromal components in PDGCAs_1 contribute to a tumor-supportive niche characterized by enhanced immune evasion and metabolic flexibility.

Tumor-associated mesenchymal stem cells promote immunosuppression in gastric cancer, often through the modulation of cytokines such as IL-6 and IL-10 [60,61]. In our study, tumor-derived stromal cells cultured in MSCM and ECM exhibited a mesenchymal-like phenotype enriched in stemness, inflammatory, and tumor progression pathways. The PDGCAs_1 model with higher MSCM-ECM stromal content showed upregulated IL-10 and IL-17 signaling, reinforcing their role in immunosuppression. The interaction between PDGCOs and stromal cells in PDGCAs_1 increased cytokine secretion, highlighting the critical role of stromal components in modeling tumor dynamics, particularly for evaluating immunotherapy responses.

Although chemotherapy remains a cornerstone in gastric cancer treatment, resistance frequently limits its effectiveness, especially in advanced stages [62]. The PDGCAs model enables patient-specific assessment of drug responses. In our study, assembloids derived from patient 1 exhibited reduced sensitivity to FLOT, FOLFIRI, and paclitaxel when co-cultured with stromal cells, suggesting a microenvironment-driven resistance mechanism. Notably, this in vitro drug resistance mirrored the patient’s clinical outcome: despite receiving multiple chemotherapy regimens—including neoadjuvant and adjuvant FLOT, followed by FOLFIRI and weekly paclitaxel—the disease progressed, and the patient succumbed within one year of surgery. This case highlights the translational relevance of the assembloid model for identifying individualized resistance phenotypes.

However, this stromal-mediated resistance was not consistently observed across all patient-derived models, indicating that tumor microenvironment effects on drug response are heterogeneous and influenced by patient-specific stromal features. Supporting this, recent clinical studies have found that stromal-enriched gastric tumors are less responsive to chemotherapy due to increased infiltration of CAFs and immunosuppressive cells [63]. Conversely, patients with low extracellular matrix risk scores show enhanced responses to chemotherapy and targeted therapies such as crizotinib, afatinib, and 5-FU [64,65,66], while those with high stromal signatures tend to respond poorly. These findings reinforce the biological plausibility of our results and emphasize the importance of incorporating stromal profiling into preclinical modeling.

Previous studies have shown that gastric cancer organoids can exhibit diverse growth patterns—such as glandular, solid, or discohesive morphologies—that are associated with tumor differentiation, molecular subtypes, and therapeutic response [7,9]. In our study, glandular organoids from patient 1 exhibited marked sensitivity to dasatinib, afatinib, and gefitinib—most of which are not FDA-approved for gastric cancer—suggesting a distinct molecular profile that may confer responsiveness to repurposed therapies. In contrast, solid organoids and assembloids from patients 2–4 were largely unresponsive, highlighting interpatient heterogeneity in treatment outcomes and the critical role of tumor-intrinsic factors in modulating drug response within the tumor microenvironment. Future studies should investigate whether specific morphological features of assembloids correlate with differential drug sensitivity, potentially offering a phenotypic marker for therapeutic stratification.

Growing evidence supports the use of small-molecule MET or ERBB2 inhibitors in gastric cancer, particularly in molecularly defined subgroups with receptor overexpression [67,68,69,70]. In this study, both PDGCOs_1 and PDGCAs_1 were sensitive to the MET inhibitor crizotinib, likely reflecting a tumor-intrinsic sensitivity in patient 1’s epithelial cells that permitted effective drug targeting, even in the presence of stromal-mediated resistance to other agents. CAFs in non-small cell lung cancer promote resistance to osimertinib through MET, Akt, epithelial-to-mesenchymal transition, and stemness pathways [71]. Consistent with these observations, stromal cells contributed to resistance against osimertinib, afatinib, and gefitinib in PDGAs_1 but not in PDGCOs_1.

Stromal interactions can both reduce drug efficacy in otherwise responsive organoids and, in some cases, help overcome intrinsic resistance. Fibroblasts have been shown to sensitize tumor cells to chemotherapy through mechanisms such as increased oxidative stress, paracrine signaling, and competition for nutrients or oxygen [72,73,74,75,76]. The presence of fibroblasts may elevate reactive oxygen species (ROS) levels within the tumor microenvironment, enhancing tumor cell sensitivity to ROS-dependent agents such as doxorubicin [77]. These findings collectively highlight the value of our personalized assembloid platform in modeling complex tumor–stroma interactions and identifying patient-specific drug sensitivities, including responses to repurposed or context-dependent agents. They also reinforce the importance of incorporating stromal components into drug screening systems.

This assembloid model presents a promising alternative to animal models, offering a more physiologically relevant, cost-effective, and ethically favorable platform for personalized oncology research [78]. The system enables the establishment of a comprehensive tumor-derived biobank containing matched epithelial and stromal cell subpopulations, providing accurate, patient-relevant data for AI -driven analysis, along with cells for functional testing. This approach may accelerate therapeutic discovery and support the optimization of personalized and combination treatments [79]. Further RNA-seq analysis of PDGCAs compared to native tissue is needed to better characterize stromal cell subtypes within assembloids. A limitation of this study is that we did not test multiple stromal ratios to assess their impact on drug response; consequently, we could not directly correlate stromal content with clinical outcomes. However, the flexibility of the assembloid system offers the potential to model varying tumor–stroma compositions and better align in vitro drug response with patient–clinical data in upcoming studies. Future work will expand this model by incorporating matched immune cells for immunotherapy assessment and further correlate experimental findings with clinical outcomes.

In summary, we developed a 3D human-derived gastric cancer assembloid model that integrates PDGCOs with matched stromal cell subpopulations cultured under different culture conditions. This model enables comprehensive genetic and gene expression profiling and functional drug testing, providing a robust system for investigating drug resistance and guiding personalized treatment strategies.

## 5. Conclusions

This study highlights the critical role of different autologous stromal cell subsets in shaping tumor behavior and modulating drug response in gastric cancer. Our findings mark an important advancement in personalized oncology and offer valuable insights into the mechanisms of drug resistance within the tumor microenvironment. By recapitulating patient-specific tumor–stroma interactions, this assembloid platform improves the physiological relevance of in vitro models and enhance the translational potential of preclinical drug testing.

## Data Availability

The genomic datasets generated in this study have been deposited in the BioSample database under the accession number SAMN41457005–10. The transcriptomic datasets generated in the study are available in the BioSample database under the accession number SAMN41539923–47. These datasets are available at the following URL: ID 1113570—BioProject—NCBI.

## Supplementary Materials

The following supporting information can be downloaded at: https://www.mdpi.com/article/10.3390/cancers17142287/s1, Figure S1. Characterization of Gastric Tumor-Derived Stromal Cell Subpopulations; Figure S2. Evaluation of Organoid and Assembloid Medium Suitability; Figure S3. Growth of Assembloids Over Time.

## Abbreviations

The following abbreviations are used in this manuscript:

CAFs	Cancer-Associated Fibroblasts
ECM	Endothelial Cell Medium
FDR	False Discovery Rate
FM2	Fibroblast Medium-2
MSCM	Mesenchymal Stem Cell Medium
PDGCOs	Patient-Derived Gastric Cancer Organoids
PDGCAs	Patient-Derived Gastric Cancer Assembloids
3D	Three-Dimensional
