# Supplementary material for: Patient-Derived Gastric Cancer Assembloid Model Integrating Matched Tumor Organoids and Stromal Cell Subpopulations
Source: Cancers (Basel). 2025 Jul 9;17(14):2287. doi: 10.3390/cancers17142287 (PMC12293640; doi:10.3390/cancers17142287)
Supplement: Supplementary file 1 [file cancers-17-02287-s001.zip › cancers-3662316-supplementary.pdf]

cancers

MDPI

Article

Patient-Derived Gastric Cancer Assembloid Model Integrating Matched Tumor Organoids and Stromal Cell Subpopulations

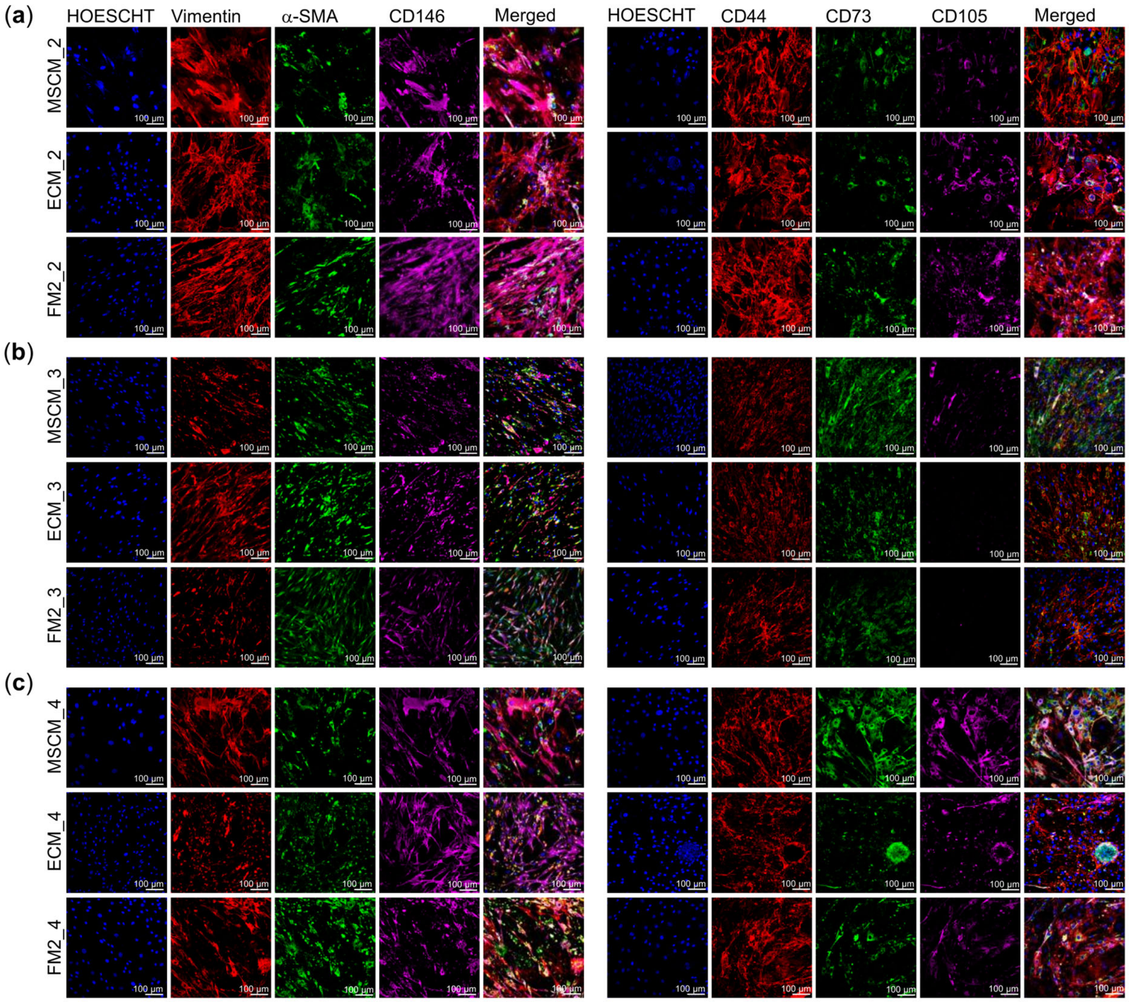

Figure S1. Characterization of Gastric Tumor-Derived Stromal Cell Subpopulations. (**a-c**) Representative immunofluorescence images of stromal cells derived from three gastric tumor samples: Sample 1(**a**), Sample 2 (**b**), Sample 3 (**c**). Cells were cultured in MSCM, ECM, or FM2 media and stained for stromal and mesenchymal markers including Vimentin/αSMA/MCAM and CD44/CD105/CD73. Nuclei were counterstained with Hoechst. Scale bar = 100 µm.

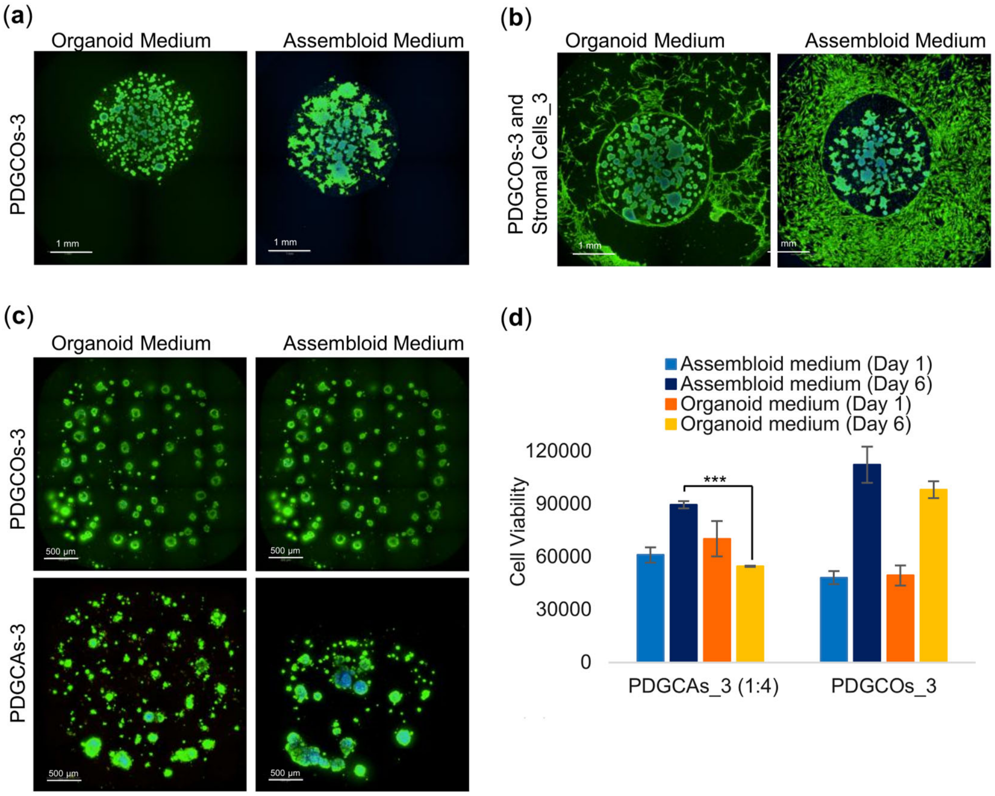

Figure S2. Evaluation of Organoid and Assembloid Medium Suitability. Representative calcein/propidium iodide images of cells cultured in organoid or assembloid medium: (**a**) Organoids embedded in matrigel domes; (**b**) Co-cultures of organoids embedded in matrigel domes with stromal cells seeded in the surrounding well; (**c**) Organoids and assembloids seeded in low-attachment plates. (**d**) Quantification of cell viability in low-attachment cultures using the CellTiter-Glo assay. Data represent mean ± SEM (n=2).

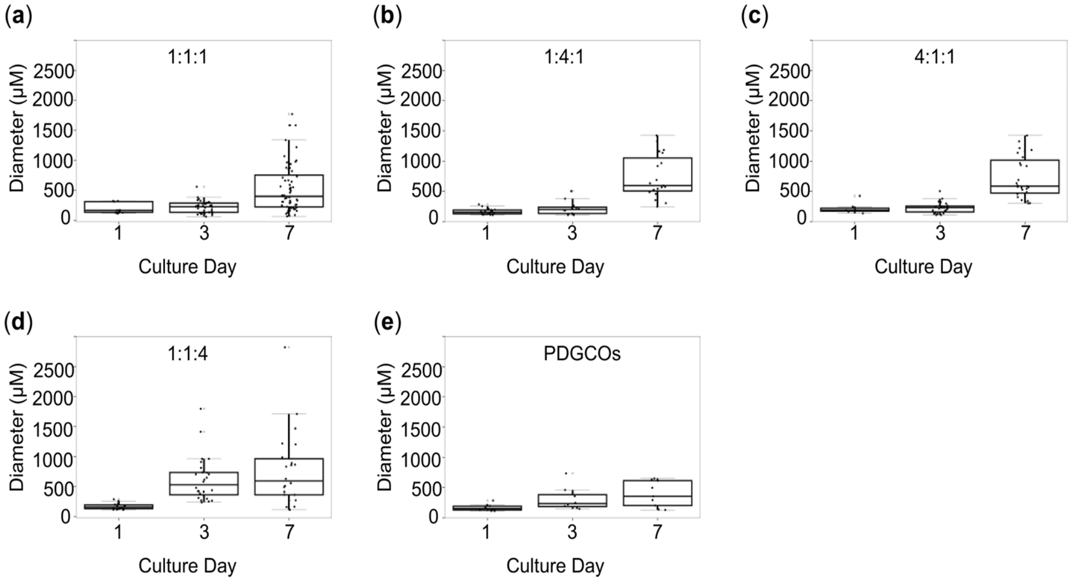

Figure S3. Growth of Assembloids Over Time. Boxplots showing changes in the size of PDGCAs_1 and PDGCOs_1 over a 7-day culture period. PDGCAs_1 were generated by combining stromal cells cultured in MSCM-ECM, FM2 and PDGCOs_1 at different ratios: (**a**) 1:1:1, (**b**) 1:4:1, (**c**) 4:1:1, and (**d**) 1:1:4. (**e**) PDGCOs_1 were included as a control. Size was evaluated by measuring the diameter of each structure over time.
